# Real-Time CGH Generation by CUDA-OpenGL Interoperability for Adaptive Beam Steering with a MEMS Phase SLM

**DOI:** 10.3390/mi13091527

**Published:** 2022-09-15

**Authors:** Chin-I Tang, Xianyue Deng, Yuzuru Takashima

**Affiliations:** James C. Wyant College of Optical Science, University of Arizona, 1630 E. University Blvd., Tucson, AZ 85719, USA

**Keywords:** LiDAR, beam steering, phase light modulator (PLM), MEMS, computer-generated hologram (CGH), GPU computing

## Abstract

Real-time, simultaneous, and adaptive beam steering into multiple regions of interest replaces conventional raster scanning with a less time-consuming and flexible beam steering framework, where only regions of interest are scanned by a laser beam. CUDA-OpenGL interoperability with a computationally time-efficient computer-generated hologram (CGH) calculation algorithm enables such beam steering by employing a MEMS-based phase light modulator (PLM) and a Texas Instruments Phase Light Modulator (TI-PLM). The real-time CGH generation and display algorithm is incorporated into the beam steering system with variable power and scan resolution, which are adaptively controlled by camera-based object recognition. With a mid-range laptop GPU and the current version of the MEMS-PLM, the demonstrated scanning speed can exceed 1000 points/s (number of beams > 5) and potentially exceeds 4000 points/s with state-of-the-art GPUs.

## 1. Introduction

Laser beam steering (LBS) by using spatial light modulators (SLMs) has been adopted for a variety of scientific and industrial optical instruments and applications such as optical tweezers, optical switches [1,2], optical communication systems, and lidar [3,4,5]. In LBS applications, computer-generated holograms (CGHs) displayed on a spatial light modulator (SLM) alter the phase and amplitude of illumination and, consequently, a diffraction pattern is manipulated. With a phase light modulator (PLM), the diffraction efficiency of a CGH for beam steering outperforms that of an amplitude-based CGH. In this regard, a phase CGH is suitable for applications with high optical throughput such as beam steering for lidar [3,4,5]. Phase-based light modulation is commonly employed by SLMs such as a Liquid Crystal on Silicon (LCoS) SLM [6]. In particular for lidar applications, the device area (A) is a critical aspect since the maximum detectable range scales with √A [7]. Despite the large device area of LC-based devices, the slow response time of liquid crystal polymers limits the speed of beam scanning (frame rate) to up to hundreds of Hz [8]. Moreover, linear polarization is required for a LCoS SLM. Due to the scattering of object surface, returning light from objects is not completely linear even when linearly polarized illumination is employed for a lidar transmitter. The polarization requirement reduces the photon throughput at least by half. The limited frame rate and polarization prohibit LC-based SLM devices from high-speed and high-efficiency beam steering applications.

A reflective and Micro Electro-Mechanical System (MEMS) SLM and PLM have recently become available [9,10]. MEMS-SLMs are uniquely positioned in terms of device area, operation speed, and diversity in polarization for a lidar transmitter and receiver. Commercially available MEMS-SLMs, such as the Digital Micromirror Device (DMD) accommodates an array area of over 140 mm^2^, operating at a tens of kHz frame rate [11]. The MEMS-PLM modulates phase by piston motion of micromirror array; therefore, no polarization specific illumination is required. Beam steering by SLMs, however, including the MEMS-PLM, suffers from a relatively narrow scanning angle, on the order of λ/d, where λ and d are the wavelength and the pixel period, respectively. Recently, over 48 degrees of angular throw by diffractive beam steering is demonstrated by employing unpolarized short-pulse illumination in a synchronous manner to the movement of the MEMS mirror array of the DMD [4,12]. Combination of two scanning modalities with pulsed illumination increases number of scanning points while not sacrificing the fast refresh rate of MEMS-SLMs [13,14]. As those works indicate, with the large Etendue (product of area and angular throw) of arrayed MEMS-SLMs, laser beam steering is feasible with a high frame rate, a wide field of view, a large device area (consequently increased range for object detection), and a lower laser power density satisfying eye safety regulation.

In addition to MEMS-SLMs’ high speed, large beam area, and large angle scanning operation, random access steering makes MEMS-PLMs even more attractive. Instead of scanning the entire field of view (FOV) in a sequential manner, beam is steered into and scans the vicinity of the object. Such random-access scanning increases the scanning rate and the number of beams/s. The other interesting use case is camera-assisted and foveated lidar. For example, positions and relative distances among multiple objects are first estimated by using a camera. Based on the estimation, the MEMS-PLM steers beams into multiple objects to measure precise distance information. The camera–lidar hybrid object detection makes the lidar system more adaptive; consequently, it solves challenges in lidars such as a strong reflection signal from retro-reflective objects, i.e., traffic signs. Additionally, the dynamic range of a lidar detector can be effectively increased by pre-adjusting the beam intensity to objects, based on the initial estimation of the relative distance of objects by camera. In this way, the signal levels returning from the multiple objects are equalized.

Foveated camera–lidar interoperability solves major challenges for lidar; however, it requires a fast and real-time calculation and display of a CGH without resorting to the iterative CGH calculation algorithm, along with interfacing the algorithm to the camera-based object detection. Such fast and non-iterative calculation of CGHs displaying simple objects such as line is reported by using a look-up table, and deep learning. For a more complex image, a single FFT-based CGH calculation is reported [15,16,17]. A real-time CGH generated for displaying a relatively complex structure is also reported [18]. Along with those works, we address the challenge in particular on CGH generation for diffractive beam steering: generating a simpler pattern, such as multiple beams while varying the beam intensity of those beams based on an input from camera. The whole process is performed in real time and satisfies the frame rate requirement of a modern lidar system.

In this paper, we address each of the building blocks of the foveated lidar framework in particular by using a recently developed high-speed phase MEMS-PLM, the Texas Instruments Phase Light Modulator (TI-PLM). In Section 2, we discuss the real-time CGH calculation algorithm for adaptive and multi-ROI (region of interest) beam steering with a variable beam ratio by GPU-accelerated CUDA-OpenGL interoperability. Benchmarking results in CGH computation and the display speed is reported. In Section 3, the integration of multiple object recognition and estimation of mutual distance by a deep learning model (YOLOv4-tiny [19]) is addressed along with beam steering by using the information. Real-time multi-point and variable beam ratio beam steering is demonstrated and discussed in Section 4. In Section 5, we address controlling the power ratio in multi-point beam steering by simulation and experiment. Finally, the limitations and scalability of the approach for lidar with adaptive laser beam steering are discussed.

## 2. Multi-Point and Variable Beam Steering Implemented in CUDA-OpenGL Interoperability with the TI-PLM

Adaptive and foveated beam tracking with the TI-PLM involves three building blocks: (1) GPU-based calculation of a CGH for multi-point beam steering, (2) CUDA-OpenGL interoperability to display a CGH on the TI-PLM, and (3) AI-based and real-time multiple object recognition by camera.

### 2.1. A CGH for Multi-Point and Variable Beam Ratio Steering

**Figure 1:**
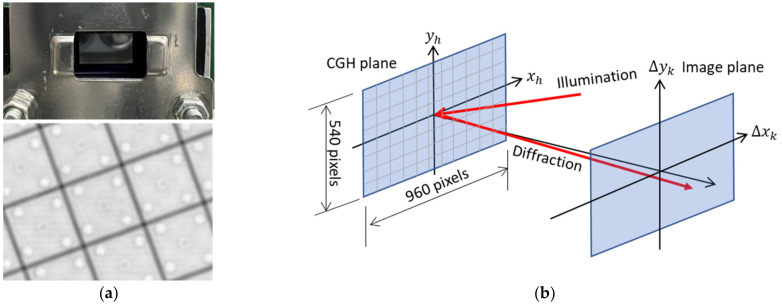
(**a**) The TI-PLM and image of pixels; (**b**) schematic diagram of the CGH plane $\left(x_{h},\;y_{h}\right)$ and the image plane $\left(\Delta x_{k},\;\Delta y_{k}\right)$. For a given beam-steering angle $\left(\Delta x_{k}/f,\Delta y_{k}/f\right)$, the phase of the pixel located at $\left(x_{h},\;y_{h}\right)$ is calculated by Equation (1).

The TI-PLM is a MEMS-based reflective phase light modulator [9,10]. The phase is modulated by a 960 × 540 pixel array of micromirrors with a pixel period d = 10.8 um with piston motion. The maximum phase modulation depth of the current generation of the PLM is 2π at 633 nm (Figure 1a).

The incident plane wave to the PLM is diffracted by the phase modulation in tilt across the PLM plane. Equivalently, a lateral shift of the focused spot is observed at the back focal plane of the lens placed between the PLM and the image plane. The lateral shift of the beam $\left(\Delta x_{k},\;\Delta y_{k}\right)$ is related to the phase of the SLM $\emptyset_{k}\left(x_{h},\;y_{h}\right)$ by,

(1) $$\phi_{k}\left(x_{h},y_{h},\Delta x_{k},\;\Delta y_{k}\;\right)=\left(\frac{2\pi }{\lambda f}\left(\Delta x_{k}x_{h}+\Delta y_{k}y_{h}\right)\right)\;mod\;2\pi$$

where $\left(x_{h},\;y_{h}\right)$ is the pixel coordinate of the SLM and $\left(\Delta x_{k},\;\Delta y_{k}\right)$ is a lateral shift of the beam with respect to the 0^th^-order beam indexed by *k* at the image plane of a focusing lens. F is the focal length of the lens. The maximum displacement $\sqrt{\Delta x_{k}{}^{2}+\Delta y_{k}{}^{2}\;}$ is limited by the wavelength λ and the pixel pitch d and is given by λf/2d.

We consider steering the beam into multiple points on the image plane while varying the power of each of the diffracted beams. Assuming a plane wave with unit amplitude illuminates the TI-PLM, the modulated field is given by,

(2) $$\psi \left(x_{h},y_{h},\Delta x_{k},\;\Delta y_{k}\;\right)=\overset{n}{\underset{k=1}{\sum }}A_{k}e^{j\phi_{k}}$$

For a phase-only MEMS-SLM, the phase *θ* of the hologram is given by,

(3) $$\theta \left(x_{h},y_{h},\Delta x_{k},\;\Delta y_{k}\;\right)=\arg\left(\;\overset{n}{\underset{k=1}{\sum }}A_{k}e^{j\phi_{k}}\;\right)$$

So far, we know the phase on the hologram plane to generate multiple points on the image plane. To decrease the computational time, Equation (3) can be re-written as,

(4) $$\theta \left(x_{h},y_{h},\Delta x_{k},\;\Delta y_{k}\;\right)=\tan^{-1}\left(\frac{\sum_{k=1}^{n}A_{k}\sin\left(\phi_{k}\right)}{\sum_{k=1}^{n}A_{k}\cos\left(\phi_{k}\right)}\right)$$

Equations (3) and (4) generate identical phase holograms. However, with Equation (4), the computational time is substantially decreased. Equation (4) indicates that phase at each pixel coordinate ($x_{h},y_{h}$) is independently calculated by summation operation. Due to the large amount of independency and low complexity in the computation of phase θ, the phase of each pixel can be processed in parallel by using CUDA (Compute Unified Device Architecture) with a GPU (Graphic Processing Unit) [20,21]. Further, a substantial part of rendering of a CGH and streaming them to the TI-PLM is also handled by the GPU by CUDA-OpenGL interoperability while applying a CGH rendering scheme specific to the TI-PLM [5,22]. In this manner, data transfer required between the CPU and the GPU is minimized; consequently, the CGH computational time and display time are drastically decreased.

### 2.2. Parallel Processing of CGH Calculation

**Figure 2:**
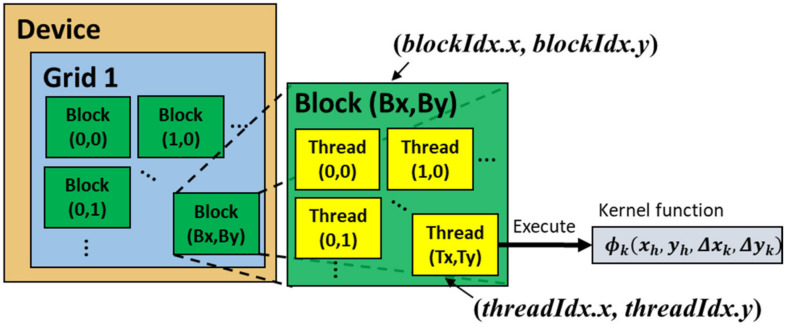
CUDA for CGH calculation. Each thread handles a pixel of a CGH, calculating the phase value using Equation (1) for single-beam steering, or Equation (4) for multi-beam steering. $\Delta x_{k}$ and $\Delta y_{k}$ are the lateral shift in x and y direction (Figure 1b), respectively.

CUDA is a parallel programming platform introduced by NVIDIA to access GPU resources by organizing threads, blocks, and grids for CUDA kernel functions. In CUDA, a grid is composed of a set of blocks, and a block is composed of a set of threads. One thread is a unit of parallel processing in the GPU that handles calculation of the phase of a single pixel (Figure 2). Since the TI-PLM has 960 × 540 physical pixels, we allocate (32, 30) threads in a single block, and (30, 18) blocks in a grid, which results in (960, 540) threads, and the CGH of (960, 540) pixel area is generated.

The pixel position ($x_{h},\;y_{h}$) and the index of the blocks and threads in a block are related by the parameter set of (threadIdx.x, threadIdx.y) as the thread index, (blockDim.x, blockDim.y) as the number of threads in a block, i.e., (32, 30) in our case, and (blockIdx.x, blockIdx.y) as the indices of the blocks. Phase values $\phi_{k}\left(x_{h},y_{h},\Delta x_{k},\;\Delta y_{k}\;\right)$ for a given ($\Delta x_{k},\;\Delta y_{k}$) is computed in a distributed manner. Computational results at each of the pixel positions ($x_{h},y_{h}$) are compiled by using indices and are given by,

(5) $$x_{h}=blockIdx.x\times blockDim.x+threadIdx.x$$

(6) $$y_{h}=blockIdx.y\times blockDim.y+threadIdx.y$$

For example, the phase at a pixel position of (102, 334) for single-beam steering is represented by $\phi_{k}\left(102,\;334,\;\Delta x_{k},\;\Delta y_{k}\right)=\left(\frac{2\pi }{\lambda f}\left(102\Delta x_{k}+334\Delta y_{k}\right)\right)\;mod\;2\pi$.

### 2.3. CUDA-OpenGL Interoperability for CGH Calculation, Rendering and Display

CUDA-OpenGL interoperability combines the advantages of GPU-based calculation and GPU-accelerated display via sharing OpenGL resources with CUDA, and mapping a buffer object from OpenGL to CUDA memory [22].

**Figure 3:**
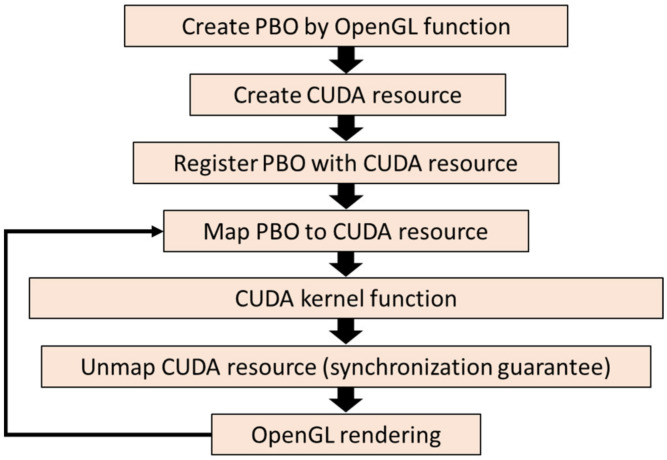
Operational flow of CUDA-OpenGL interoperability.

**Figure 4:**
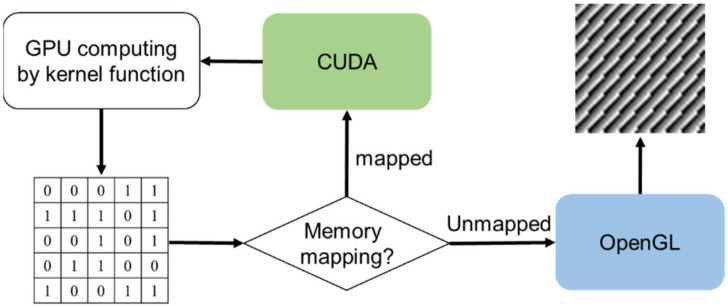
Schematic diagram for CUDA-OpenGL interoperability. CUDA and OpenGL share the same memory by mapping the buffer with CUDA. Once it has unmapped the buffer, OpenGL can directly render the calculated CGH.

To implement CUDA-OpenGL interoperability, the CUDA resource should share the memory with a pixel buffer object created by OpenGL. In Figure 3, the operational flow is listed. First, we declare global variables that will be used to store handles to the data we intend to share between OpenGL and CUDA, and then initialize the OpenGL library (GLUT) and create a graphics window. The pixel buffer object (PBO) stores the pixel data and asynchronously transfers the pixel data to the graphic card without wasting CPU cycles. Next, we register the PBO with the CUDA resource to share the buffer with both OpenGL and CUDA drivers. Then, we map the buffer to CUDA memory, meaning pointing the pointer of CUDA memory to the OpenGL buffer. Next, we use CUDA to calculate the pixel data through the kernel function and store the mapped memory so that OpenGL can render the results directly once the mapping between CUDA and the buffer is cancelled as well as mapping the buffer to CUDA to keep processing until the next frame is initiated (Figure 4). The workflow minimizes data transfer between the CPU and the GPU and maximizes the throughput of CGH calculation.

## 3. Multi-Point and Real-Time Beam Tracking System with Camera-Based Adaptive Beam Steering and Pre-Estimation of the Position and Size of the Target

**Figure 5:**
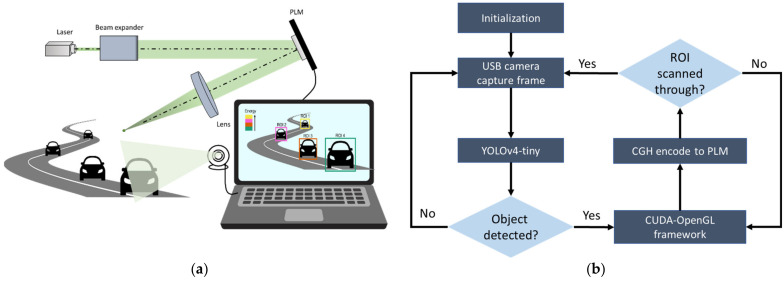
(**a**) The system of adaptive beam steering. Multiple regions of interest (ROIs) are captured by camera. (**b**) Workflow of adaptive beam steering. Camera-based rough order of magnitude (ROM) detection of the relative size of objects. For example, the relative appearance of multiple cars indicates the relative distance of multiple ROIs.

CUDA-OpenGL interoperability enables fast calculation of a CGH based on real-time input, i.e., camera-based object detection (Figure 5). First, the camera captures multiple objects followed by identification of the position and the extent of the multiple objects within an FOV (Figure 5a). The task, defining the region of interest (ROI), is performed by a YOLOv4-tiny pretrained model for object recognition [19]. The camera captures the scene and input to the pretrained deep learning model. When the object of interest is detected, the coordinates and the extent of ROIs will be assigned to GPU-based CGH processing. The calculated CGH is displayed on the TI-PLM through the HDMI. The camera will capture the next frame once the objects of interest in the previous scene are scanned through. In this manner, a CGH simultaneously steers beams into multiple ROIs that are calculated and displayed on the TI-PLM. Furthermore, with Equation (4), it is capable of controlling the beam energy distribution to equalize the returning signal strength by assuming that the ratio of the apparent extent of objects depends on distance. Within the ROIs, objects are sequentially scanned while allocating appropriate beam power to each of the ROIs.

## 4. Experimental Results

**Figure 6:**
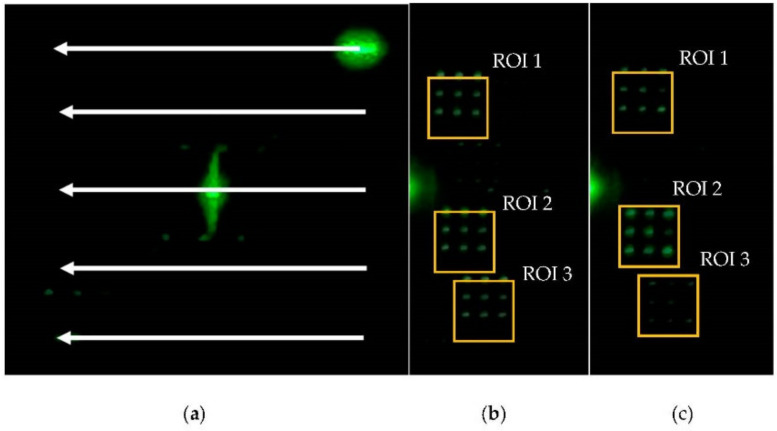
(**a**) Single ROI scan with single-beam steering. See Video S1. (**b**) Multiple ROIs scan with the same beam weights. See Video S2. (**c**) Multiple-ROI scan with different beam weights (ROI 2 > ROI 1 > ROI 3). See Video S3.

Based on the camera input, adaptive beam steering steers the beam into a single ROI (Figure 6a), multiple ROIs (Figure 6b), and multiple ROIs with a variable beam ratio (Figure 6c). CGH calculation time and beam steering are demonstrated. Benchmarking and beam steering are performed by using a laptop with a NVIDIA GeForce GTX 1650-ti GPU (16 Streaming Multiprocessors with the total of 1024 CUDA cores), an Intel© C©TM) i7-10750H CPU and 16 GB memory. CUDA version 11.4(NVIDIA) was used.

### 4.1. Benchmarking of CGH Calculation Time: On a Laptop

The TI-PLM supports two data transfer modes, the monochromatic and RGB modes. The monochromatic mode displays one encoded CGH for each of the frames at a maximum frame rate of 60 Hz. In contrast, the RGB mode transfers three CGHs in 60 Hz such as a color-encoded CGH in 60 Hz that increases the display frame rate to 180 Hz. Table 1 and Table 2 tabulate the CGH calculation speed performed on the laptop using the monochromatic (1 CGH/frame) and RGB (3 CGHs/frame) modes for single- and multi-beam steering. Both the CPU and GPU GCH processing programs are written in C++. The CPU code is based on OpenCV, a well-optimized image processing library; the GPU code is based on the CUDA-OpenGL approach. As the tables show, a GPU performs 3.5- and 7.8-fold faster than CPU processing for the monochromatic and RGB modes, respectively, in single-beam steering. For multi-beam steering, the GPU speeds up to 6.8- to 10.8-fold (monochromatic mode) and 10.4- to 15-fold (RGB mode) faster from 2-beam to 7-beam steering. The following beam-steering speed test experiments (laptop with a PLM) are using the GPU-RGB mode since it performs the best.

### 4.2. Benchmarking of CGH Calculation Time: Laptop with a PLM

As Table 2 shows, the GPU-based CGH calculation time (without a PLM connected) from single-beam steering to 5-beam steering performs above 60 FPS (180 CGHs/s). With a PLM connected over HDMI (Table 3), the PLM frame rate is now limited to 180 FPS (180 CGHs/s) and is limited by the data rate of the current PLM driver. While steering and scanning multiple beams over multiple ROIs, the effective scanning speed is enhanced; however, such multi-point CGH calculation imposes an overhead. Table 3 summarizes the calculation speed and the scan speed in the RGB transfer mode for scanning 1, 2, 3, 4, 5, 6, and 7 ROIs. As Table 3 shows, the frame rate of the PLM slightly decreases as the number of beams (or number of ROIs) exceeds 6; however, the increased number of scanning points for multiple ROIs still improves the overall scan rate. For example, 5 independent ROIs are scanned with 180 points/s for each of the ROIs, results in an effective beam-steering speed of 900 points/s.

### 4.3. Multi-Point and Adaptive Beam Tracking with a Variable Beam Ratio

**Figure 7:**
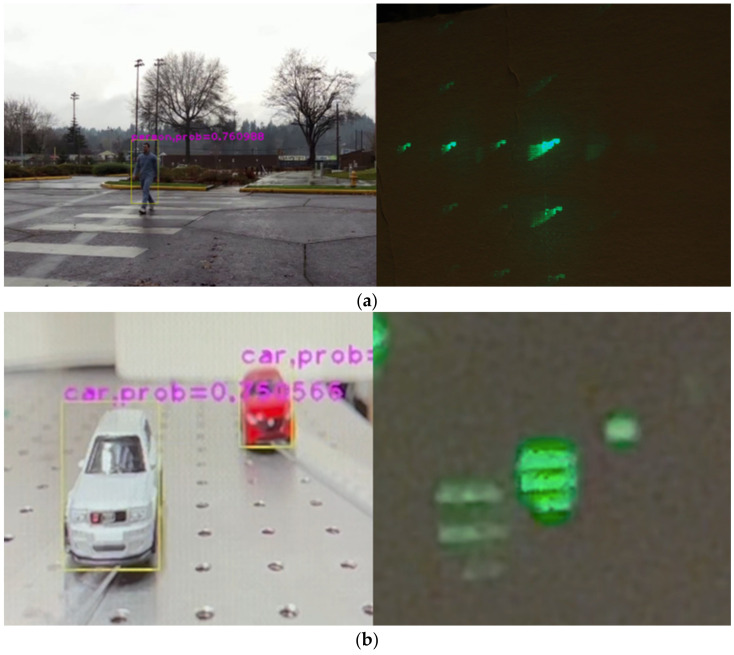
Adaptive single-beam steering demo. The USB camera captures the image of (**a**) a pedestrian crossing the street on an LCD screen. The TI-PLM scans through the man as he is moving. See Video S4. (**b**) Two miniature cars captured by a USB camera. A distant (smaller in appearance) car is steered with a higher-energy beam; Closer (larger in appearance) car is steered with a lower-energy beam. See Video S5.

With a camera connected, adaptive single- and multi-beam tracking is demonstrated by capturing an image of a pedestrian displayed on an LCD monitor (Figure 7a) and two miniature toy cars (Figure 7b). Based on the captured video, the extent of ROIs is calculated in real time with YOLOv4-tiny. Since these two cars have approximately the same size, the ratio of the distance of the cars is approximately equal to the ratio of the extent of the ROIs of each of the cars. In such a case, the beam intensity ratio was modulated as the inverse of the ratio of the extent of each ROI, so that returning signal levels from each of the ROIs are equalized. The weighting factor $A_{k}$ for the k-th ROI is given by,

(7) $$A_{k}=\frac{C_{k,\;\bar{k}}}{\sqrt{H_{k}\times W_{k}}}$$

where $H_{k}$ and $W_{k}$ are the height and the width of the k-th ROI, $\bar{k}$ is the index of ROIs other than the k-th ROI, and $C_{k,\;\bar{k}}$ is a correction factor for (a) converting the amplitude to the power ratio while encoding an amplitude + phase CGH as a phase-only CGH, and (b) variation of the diffraction efficiency as a function of set ROIs. The calculation and experimental verification of the ratio of the weighting factor $A_{k,\;\bar{k}}=A_{k}/A_{\bar{k}}$ to the power ratio in two ROIs is $P_{k,\;\bar{k}}=P_{k}/P_{\bar{k}}$, where $P_{k}$ is the power to k-th ROI, is addressed later is Section 5. For the single-beam steering, the rectangular area will be scanned in real time at the maximum speed of the current generation of the TI-PLM, which is 180 points/s. For multi-point beam steering, two ROIs are scanned while adaptively varying the beam power ratio.

## 5. Calibration of the Power Ratio for Multiple ROIs and Adaptive Beam Steering

The adjustment of the beam power is performed via weighing factor $A_{k}$ (Equation (8)). In Equation (2), $A_{k}$ is defined as an amplitude of fields for the k-th ROI; however, since the TI-PLM is a phase-only SLM, the ratio of powers is not simply a square of the ratio of amplitude since the amplitude and the phase over the PLM plane are encoded as a phase-only CGH. Additionally, the TI-PLM is a pixelated SLM with a finite number of, 16, phase levels with a pixel period d = 10.8 um. The available 16 phase levels are not evenly distributed but rather non-equally between 0 and 2π [9,10]. The discretized and non-equally distributed finite levels affect the beam ratio via variation of the diffraction efficiency as a function of beam-steering angles, or the effective grating periodicity of the CGH [5]. Those effects are combined into a single correction factor $C_{k,\;\bar{k}}$.

### 5.1. Beam Ratio Experiment

**Figure 8:**
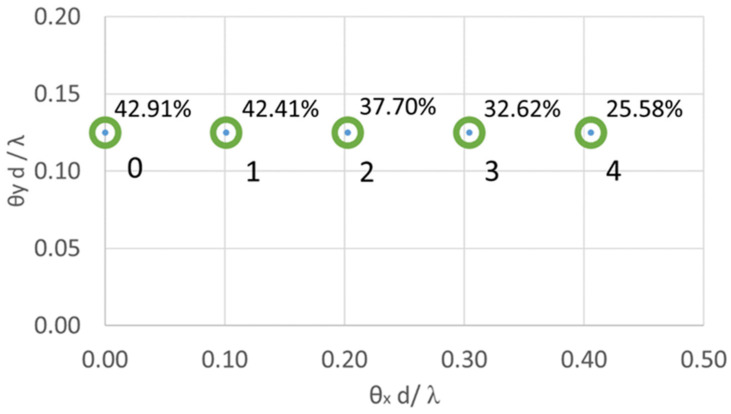
ROIs defined to characterize $P_{i,j}$ and the diffraction efficiency for k = 0–4.

To adjust the beam power ratio among multiple ROIs, we experimentally evaluated the diffraction efficiency (DE) of the TI-PLM for a case where two ROIs are simultaneously scanned at a wave length λ = 532 nm and correlated the ratio of the weighting factor, $A_{i,j}=A_{i}/A_{j}$, to the measured power ratio, $P_{i,j}=P_{i}/P_{j}$. We setup five representative diffraction angles corresponding to i=1~5, j<i (Figure 8). Beams are steered into two ROIs out of the five total ROIs. Power at i-th ROI was measured by a power meter placed at the back focal point of the f = 300 mm lens to calculate DE.

In Figure 8, the *x*-axis is the diffraction angle $\theta_{x}$ in the x-direction normalized by the wavelength λ and the pixel pitch d of the PLM, which is also represented as the inverse of the x-direction grating period $\Lambda_{x}$ in the unit of 1/pixel. This is similar for the *y*-axis. The beam index k = 0 to 4 spans and corresponds to 1/$\Lambda_{x}$ of 0 to 0.41 (1/pixels) with the same 1/$\Lambda_{y}$ of 0.125 (1/pixels). As reported by Deng et al., the diffraction efficiency monotonically decreases as the periodicity of the CGH decreases (Figure 8) [5]. By measuring the single-beam steering diffraction efficiency at those five diffraction angles, we can also evaluate the energy loss from multi-beam steering.

**Figure 9:**
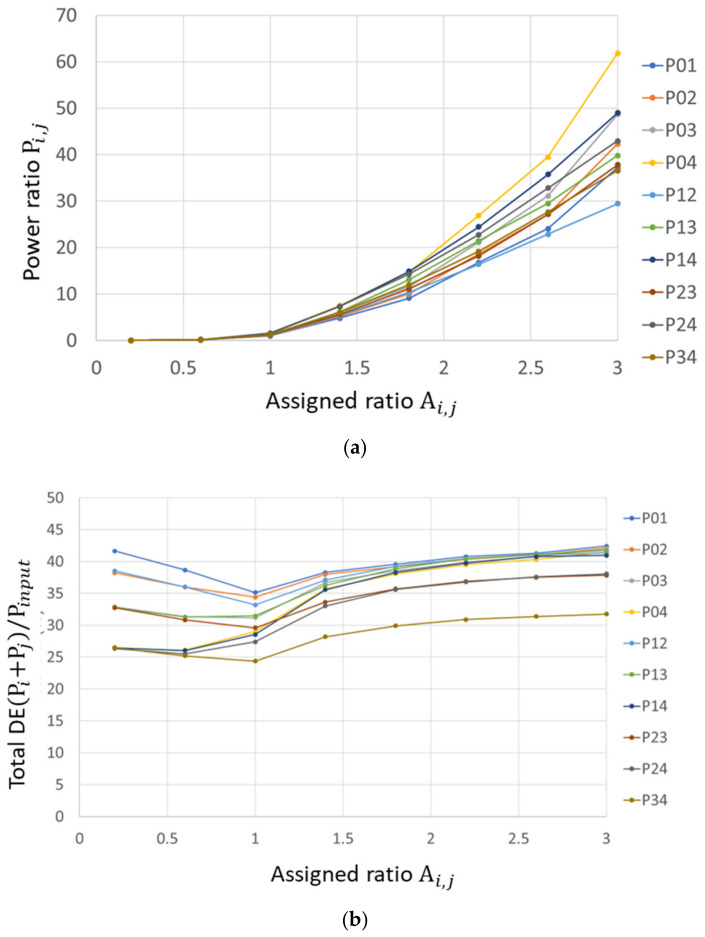
(**a**) $P_{i,j}$ as a function $A_{i,j}$ for all the combinations of (i,j), 0≤i,j≤4; (**b**) the total diffraction efficiency ${\mathrm{DE}}_{total,\;i,j}=(P_{i}+P_{j})/P_{input}$, where $P_{i}$ and $P_{input}$ are the diffracted power to i-th ROI and total power impinging upon the PLM, respectively.

Figure 9a plots $P_{i,j}$ as a function, $A_{i,j}$, for all the combinations of (i,j), where 0≤i j≤4. Figure 9b plots the total diffraction efficiency ${\mathrm{DE}}_{total,\;i,j}=(P_{i}+P_{j})/P_{input}$, where $P_{i}$ and $P_{input}$ are the diffracted power to i-th ROI and total power impinging upon the PLM, respectively. The power ratio $P_{i,j}$ and assigned weight ratio $A_{i,j}$ are well correlated, while the correlation is ROI (diffraction angle) dependent. As Figure 8 shows, the larger diffraction angle produces the lower diffraction efficiency [5]. Consequently, when steering the beam to two different angles with the assigned ratio $A_{i,j}$ = 1, the power ratio should not be 1; instead, it is affected by the ratio of DE of a single beam. Furthermore, as the beam angle separation becomes larger, a higher power ratio is observed since the difference in DE becomes larger. For example, we observed $P_{0,4}>P_{0,3}>P_{0,2}>P_{0,1}$ for $A_{i,j}=3$. This can be explained by the denominator ($P_{j}$) of the power ratio $P_{i,j}=P_{i}/P_{j}$, becomes larger due to the small diffraction angle. Figure 9b plots ${\mathrm{DE}}_{total,\;i,j}$ as a function of $A_{i,j}$ and no significant energy loss is observed for multi-beam steering compared with single-beam steering.

### 5.2. Simulation

**Figure 10:**
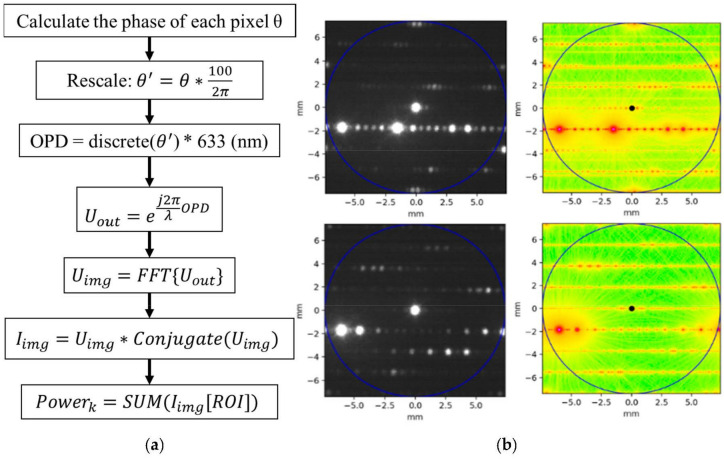
(**a**) Multi-beam steering power simulation process based on the FFT method; (**b**) the simulated intensity distribution in log scale, steering to positions 1 and 4 with the assigned weight ratio of 1, and beam steering to positions 3 and 4 with the assigned ratio of 0.6, with their experiment beam-steering image aside.

We numerically related $P_{i,j}$ to $A_{i,j}$ by simulation. The simulation flow is shown in Figure 10a. Based on Equation (4), the phase of each pixel is forced to the available 16 phase levels of the PLM to model a OPD (Optical Path Difference) map of the TI-PLM [5]. The field on the image plane is calculated by the Fast Fourier Transform of $e^{j\frac{2\pi }{\lambda }OPD}$, and the intensity distribution is the field multiplied by its complex conjugate. The power ratio, $P_{i,j}$, is calculated by integrating the intensity at the vicinity of the spots. The simulation intensity distribution results are shown in Figure 10b.

**Figure 11:**
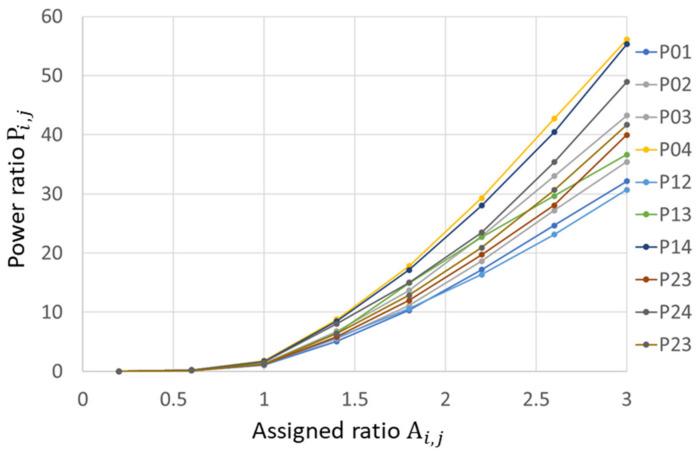
Power ratio simulation for ten cases. The simulation results also show that larger-diffraction-angle separation causes the larger power ratio.

**Figure 12:**
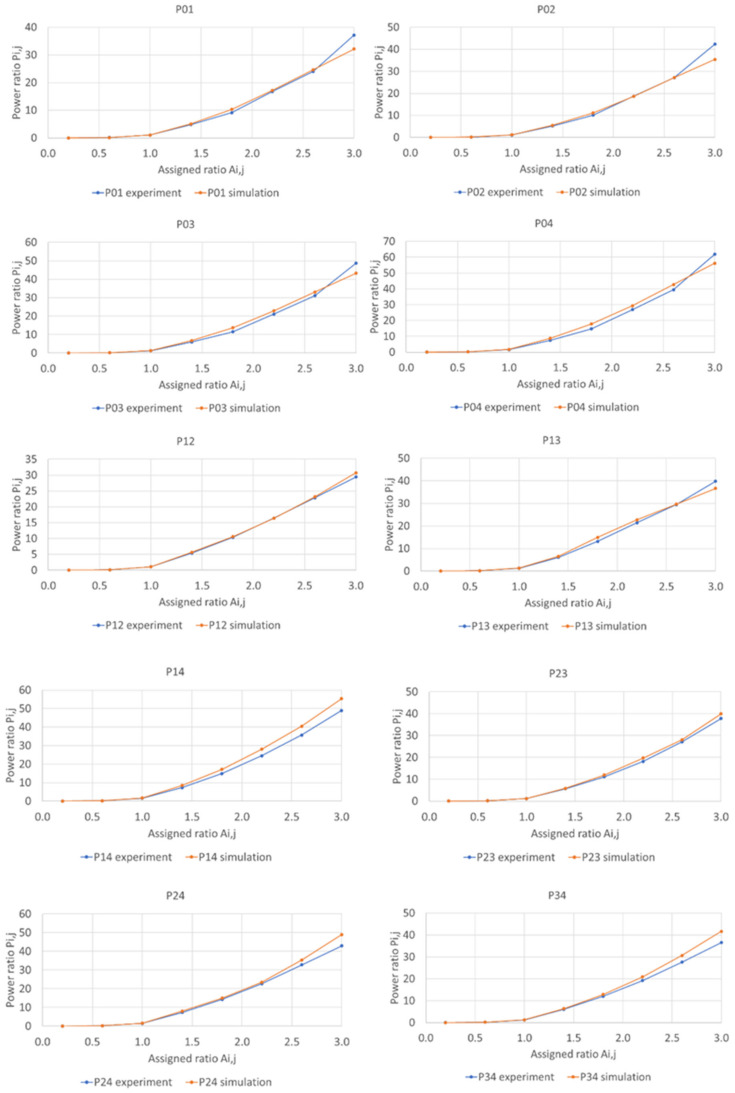
The comparison between power ratio measurement results (**blue**) and simulated power ratio results (**orange**) case by case from $P_{0,1}$ to $P_{3,4}$.

Figure 11 shows the calculated power ratio $P_{i,j}$ as a function of assigned weight, $A_{i,j}$. The results indicate that larger-diffraction-angle separation of two beam causes the larger power ratio. For instance, $A_{i,j}=3$ results in $P_{0,4}$ > $P_{0,3}$ > $P_{0,2}$ > $P_{0,1}$, and $P_{1,4}$ > $P_{1,3}$ > $P_{1,2}$. The experimental and simulated $P_{i,j}$ (Figure 9a and Figure 11) match very well, and they are also separately compared case by case in Figure 12.

## 6. Discussions

The proposed real-time CGH calculation and display by CUDA-OpenGL interoperability along with object recognition enables foveated and variable power ratio beam tracking. Two major benefits from the framework in lidar applications are (a) an improved scanning speed by avoiding raster scan, and (b) equalization of the power of returning signals from multiple objects at different distances. The pre-calculated weighting factor, $A_{i,j}$, based on object recognition enables adaptive control of the transmitter power towards multiple objects for the purpose of equalizing returning signal levels at the receiver.

**Figure 13:**
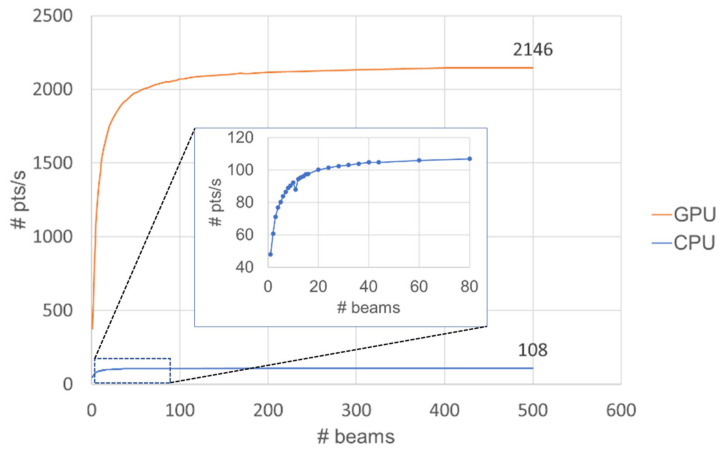
Effective beam-steering speed (RGB mode) on a GPU and a CPU. *X*-axis represents the number of points/CGH for beam steering; *Y*-axis represent the effective beam-steering speed. The inset shows the calculation speed of the CPU-based approach for the # of beams/CGH up to 80.

The following question arises: what is the optimum number of beams/CGH for multi-beam steering? The effective beam-steering speed is tested by varying the number of scanning points/CGH to determine the optimal effective beam-steering speed on a GTX-1650-ti compared with a CPU (i7-10750H). As Figure 13 shows, the effective steering speed on the GPU rapidly increases from 1 to 50 beam steering and the speed starts converging at 400 beams/CGH, which corresponds to 2146 pts/s. In contrast, the effective beam-steering speed of the CPU slightly increases up to 20 beams/CGH and it saturates at approximately 80 beams/CGH, which corresponds to 108 pts/s, which the GPU performs almost 20-fold faster than the CPU. A realistic beam-tracking scenario assisted by camera, with a large number of beams/CGH, computational architecture to detect and classify large number of objects in conjunction with CUDA-OpenGL-based calculation of a CGH, needs to be determined as a future work. Nonetheless, a current mid-range GPU, with the CGH calculation speed, can still increase the number of scanning points (or ROIs) for detection of multiple objects with beam tracking by the TI-PLM.

Currently, we have demonstrated that GPU (GTX 1650-ti, Nvidia)-based TI-PLM single-beam steering and multi-beam steering (number of beams < 6) with a speed of 180 FPS are limited by the frame rate of the current generation of the TI-PLM. When the beam-steering speed needs to be faster, three aspects can be considered. Using the higher-performing GPU increases the calculation speed of CGHs significantly. For example, the Nvidia RTX 3080 GPU (clock speed of 1440 MHz, floating-point performance of 29.77 TFLOPS, texture rate of 465.1 GTexels/s, effective memory clock speed of 19,000 MHz, memory bandwidth of 760 GB/s, 10 GB of VRAM, and 320 bits memory bus width) can perform at least 4-fold faster than the GTX 1650-ti.

The current generation of the TI-PLM supports displaying CGHs up to 180 fps, so either increasing the supported frame rate or encoding more CGHs inside a single frame will improve the beam-steering speed. The newer version of the 0.67 TI-PLM supports a 24 bit RGB mode and can transfer 24 CGHs with a frame rate of 60 FPS, or 1440 (24 × 60) CGHs can be displayed per second. The current HDMI 2.1 can handle up to 240 Hz when using a 1080 p (1080 × 1920) resolution. With a high-end and state-of-the-art GPU, a 0.67 inch PLM, and a 24 bit multiplexed CGH transfer via HDMI 2.1, it is estimated that the single-beam-steering speed can achieve 5760 points/s, and multi-beam steering can reach even more higher steering efficiency.

The angular extent of steering is still limited to several degrees due to the large pixel period of the TI-PLM compared to the wavelength. The limited angular extent can be increased to tens of degrees by employing additional MEMS-SLMs such as the DMD [11] while preserving the high-speed, foveated, and adaptive nature of the proposed beam steering by the TI-PLM. In particular, the combination of two kinds of MEMS-SLMs, the DMD and the PLM, enables rejection of the 0th-order beams as well as unnecessary diffractions (side lobes) other than the beam of interest [13]. The hybrid MEMS beam-steering optical architecture with real-time CGH generation and display by CUDA-OpenGL interoperability enables DC- and side-lobe-free adaptive beam tracking.

Simultaneous control of the power ratio requires a lookup table to determine the assigned ratio in calculating CGH with a desired power ratio. Our simulation results show good agreement between the calculated power ratio and the assigned weight ratio for beam steering with two ROIs. For three or more ROIs, an FFT-based simulation we presented here is applied to identify an empirical transfer function to determine the assigned ratio to achieve the desired power ratio among multiple ROIs.

## 7. Conclusions

CUDA-OpenGL interoperability demonstrated for real-time beam tracking with a MEMS-based phase light modulator and a Texas Instruments PLM enables real-time calculation of phase CGH and steering the laser beam into multiple objects on the fly while varying the beam power ratio. The CGH calculation method is interfaced to camera input and AI-based recognition of the position and the size of multiple regions of interest (ROIs). The end-to-end demonstration of beam tracking makes a lidar system more adaptive and intelligent, while not adopting time-consuming raster scanning. In particular, the correlation between the beam power ratio and the weight factor ratio in CGH calculation is experimentally confirmed and well predicted by simulation. The correlation factor is used as a look-up table to precisely control the beam ratio.

With the current state of the art of the GPU, the proposed CUDA-OpenGL interoperability framework for real-time CGH calculation enables adaptive, variable beam ratio and multiple-ROI tracking with over 4000 points/s of beam-steering speeds, while employing a commercial USB camera and computational devices.

## Figures and Tables

**Table 1:** CGH processing time for single-beam steering using a CPU and a GPU in the monochromatic mode (Beam # refers to number of beams; # pts/s refers to the number of points are steered per second).

Mono	GPU		CPU		Speedup Factor
Beam #	FPS	# pts/s	FPS	# pts/s	
1	232	232	67	67	3.5
2	198	396	29	58	6.8
3	184	552	23	69	8.0
4	173	692	20	80	8.7
5	161	805	17	85	9.5
6	149	894	14	84	10.6
7	140	980	13	91	10.8

**Table 2:** CGH processing time for single-beam steering using a CPU and a GPU in the RGB mode.

RGB	GPU		CPU		Speedup Factor
Beam #	FPS	# pts/s	FPS	# pts/s	
1	125	375	16	48	7.8
2	94	564	9	54	10.4
3	85	765	7	63	12.1
4	77	924	6	72	12.8
5	71	1065	5	75	14.2
6	65	1170	5	90	13.0
7	60	1260	4	84	15.0

**Table 3:** Experimental beam-steering speed in the GPU-RGB mode with a PLM connected to a laptop.

# of ROIs	With a PLM Connected	
	PLM FPS	pts/s
1	180	180
2	180	360
3	180	540
4	180	720
5	180	900
6	174	1044
7	159	1113

## Data Availability

Data are available upon request from the corresponding author.

## Supplementary Materials

The following supporting information can be downloaded at: https://www.mdpi.com/article/10.3390/mi13091527/s1, Video S1: Single ROI scan with single-beam steering; Video S2: Multiple ROIs scan with the same beam weights; Video S3: Multiple-ROI scan with different beam weights; Video S4: Pedestrian crossing the street on an LCD screen—the TI-PLM scans through the man as he is moving; Video S5: Two miniature cars captured by a USB camera—the distant (smaller in appearance) car is steered with a higher-energy beam; Closer (larger in appearance) car is steered with a lower-energy beam.
